# Immobilization of Lipase B from *Candida antarctica* in Octyl-Vinyl Sulfone Agarose: Effect of the Enzyme-Support Interactions on Enzyme Activity, Specificity, Structure and Inactivation Pathway

**DOI:** 10.3390/ijms232214268

**Published:** 2022-11-17

**Authors:** Priscila M. P. Souza, Diego Carballares, Luciana R. B. Gonçalves, Roberto Fernandez-Lafuente, Sueli Rodrigues

**Affiliations:** 1 Departamento de Biocatálisis, ICP-CSIC, Campus UAM-CSIC, 28049 Madrid, Spain; 2 Food Engineering Department, Federal University of Ceará, Campus do Pici, Bloco 858, Fortaleza CEP 60440-900, CE, Brazil; 3 Chemical Engineering Department, Federal University of Ceará, Campus do Pici, Bloco 709, Fortaleza CEP 60440-900, CE, Brazil; 4 Center of Excellence in Bionanoscience Research, Member of the External Scientific Advisory Academics, King Abdulaziz University, Jeddah 21589, Saudi Arabia

**Keywords:** heterofunctional supports, enzyme inactivation, tuning enzyme specificity

## Abstract

Lipase B from *Candida antarctica* was immobilized on heterofunctional support octyl agarose activated with vinyl sulfone to prevent enzyme release under drastic conditions. Covalent attachment was established, but the blocking step using hexylamine, ethylenediamine or the amino acids glycine (Gly) and aspartic acid (Asp) altered the results. The activities were lower than those observed using the octyl biocatalyst, except when using ethylenediamine as blocking reagent and *p*-nitrophenol butyrate (*pNPB*) as substrate. The enzyme stability increased using these new biocatalysts at pH 7 and 9 using all blocking agents (much more significantly at pH 9), while it decreased at pH 5 except when using Gly as blocking agent. The stress inactivation of the biocatalysts decreased the enzyme activity versus three different substrates (*p*NPB, S-methyl mandelate and triacetin) in a relatively similar fashion. The tryptophane (Trp) fluorescence spectra were different for the biocatalysts, suggesting different enzyme conformations. However, the fluorescence spectra changes during the inactivation were not too different except for the biocatalyst blocked with Asp, suggesting that, except for this biocatalyst, the inactivation pathways may not be so different.

## 1. Introduction

Enzymatic biocatalysis is regarded as a powerful tool to solve the demands of more complex products produced under environmentally friendly conditions [1,2,3,4]. Their high activity under mild conditions (room temperature, atmospheric pressure and aqueous medium), selectivity (discriminating between different possible products) and specificity (discriminating between different substrates) make enzymes almost ideal industrial catalysts [1,2,3,4]. However, enzymes have been developed by nature to fulfill their physiological requirements and that causes many of their features to be inadequate for industry [5]. That way, enzyme stability may not be high enough for industrial purposes; activity, selectivity and specificity have been developed for their physiological substrates, which in many instances are different from the industrially interesting ones. Even the target reactions may be different, e.g., hydrolases are used in many instances as transferase-like enzymes in kinetically controlled processes [6]. Fortunately, the tools to improve enzyme features have experienced an impressive development in the last years (metagenomics [7,8], directed mutagenesis [9,10], directed evolution [11,12], etc.). Nowadays, enzymes bearing several active centers may be developed [13], plurizymes being the most outstanding example [14,15,16].

In this context, enzyme immobilization was developed to solve the problem of enzyme recovery and reuse [17,18]. Nowadays, a proper immobilization protocol is expected to improve some other enzyme features, such as stability, activity, specificity or selectivity, enlarging the operational conditions window [19,20,21,22,23,24]. It is even possible to couple enzyme immobilization with enzyme purification, with the saving of costs and time that this implies [25].

Lipases are among the most used enzymes, both academically and in industry, due to their wide specificity, ability to catalyze many different reactions (including promiscuous ones [26,27,28], stability in diverse media [29,30,31,32] and lack of cofactors. They have the so-called interfacial activation as a common feature, which causes the open form of lipases to become strongly adsorbed on hydrophobic surfaces formed by their natural substrates (drops of oils) [33,34,35,36,37]. Lipases can be interfacially activated by any other hydrophobic surface, such as the open form of another lipase [38,39], a hydrophobic biomacromolecule [40,41] or a hydrophobic support [42]. In fact, the use of hydrophobic supports permits the one-step immobilization, purification, hyperactivation and stabilization of lipases and has become one of the most popular methods to immobilize them [43]. However, despite presenting many advantages, it also has a potential drawback. Immobilization on these supports is reversible, based on many weak hydrophobic interactions. This enables the support to be reutilized after releasing the inactivated enzyme but also poses a risk during operation: the uncontrolled release of the enzyme [43]. Although adsorption is very strong, it has been shown that the immobilized lipase molecules can passively migrate on the support surface [44] and that they can be released if submitted to drastic conditions or substrates with detergent potential [45].

To overcome this, heterofunctional acyl chemically reactive or ionic supports have been developed [46,47,48,49,50,51,52,53,54,55]. Among them, the acyl-vinyl sulfone supports [56] proved to be the most effective due to some advantages of this chemically reactive group: long spacer arm, capacity to react with diverse moieties of an enzyme (e.g., phenol, primary amino, imidazole, thiol) and the production of stable bonds [57]. Their utilization, to be compatible with the advantages of immobilization via interfacial activation, requires a first immobilization at pH 5 (to decrease the enzyme-support reactivity), followed by an incubation at higher pH to favor this support-enzyme reactivity [56]. Finally, the remaining vinyl sulfone groups in the support must be blocked [56]. This can be performed with different moieties and opens up the possibility of co-immobilizing enzymes by a different immobilization cause (even using mono-functional vinyl sulfone supports) and to tune the enzyme-support interactions [58,59,60]. This has been found to determine the enzyme catalytic features. This way, activity, specificity and stability are deeply dependent on the blocking agent. It has been recently shown, using the lipase from *Thermomyces lanuginosus*, that the blocking agent can alter the enzyme structure and even the inactivation pathway by altering the enzyme support-interactions [61]. This immobilization strategy solves the problem of enzyme release and also prevents the possibilities of acyl passive migration [44].

In this new paper, we extend the studies to the immobilization of lipase B from *Candida antarctica* (CALB) on this support. This is perhaps the most popular lipase [62,63], and its most utilized commercial immobilized form, Novozyme 435, is based on the enzyme interfacial activation on a mildly hydrophobic support [64]. However, the enzyme has a very small lid [65,66]. Thus, the increase in enzyme activity after interfacial activation is not significant. CALB immobilization on octyl-vinyl sulfone on this support has not been reported to date, and this is the objective of this report, focusing on the effect of the blocking agent in the enzyme structure (using fluorescence spectra) and the functional properties of the enzyme.

## 2. Results and Discussion

### 2.1. Immobilization of CALB on Octyl and Octyl-VS Supports

CALB was immobilized using 1 mg of enzyme per g of both supports. The loading was selected to prevent substrate diffusional limitations [67,68] or enzyme–enzyme interactions that could alter the results [69,70,71]. The immobilization was performed at pH 5 to ensure that the first step was via interfacial activation, as at pH 5 the reactivity of the reactive groups of the enzymes with the support is quite low [57]. The further incubation at a higher pH value may enable the later reaction between groups of the enzyme and the vinyl sulfone groups.

**Figure 1:**
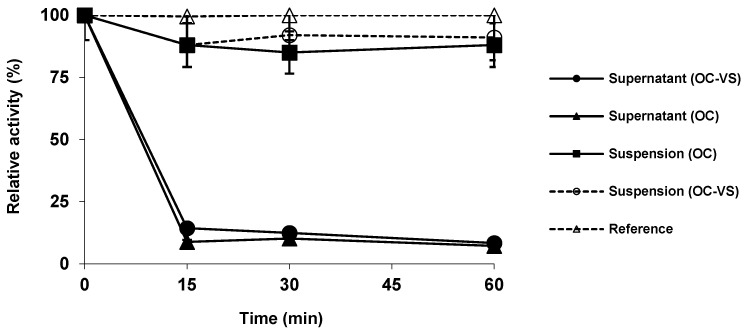
Immobilization courses of CALB (1 mg/g) on octyl (dotted line and empty symbols) and octyl-VS (solid line and symbols) beads at pH 5 and 25 °C. Experiments were performed as described in Methods.

The immobilization courses are shown in Figure 1. Both immobilizations were fast [72], and the activity of the supernatant rapidly decreased, while the reference enzyme solution activity maintained its activity intact during all immobilization time. CALB decreased its activity by around 10% after immobilization on both supports. In general, lipases increase their activity when immobilized on octyl agarose [42,43]. However, this did not happen with CALB, which decreases the expressed activity due to its small lid that does not fully isolate the active center from the reaction media [65].

After the immobilization, the biocatalyst octyl-vinyl sulfone-CALB incubated in 50 mM sodium bicarbonate at pH 8.0 and 25 °C for 4 h to favor the enzyme-support covalent reaction [56]. Table 1 shows that part of enzyme activity lost in the immobilization could be recovered by incubation at pH 8. It has been reported that this increase in enzyme activity may be explained by the conformational changes produced by the covalent attachment of the enzyme to the support that, in this case, results in changes that presented positive effects on enzyme activity [58,60].

### 2.2. Blocking Effect on Octyl-VS-CALB Biocatalysts

After the formation of covalent bonds on OC-vinyl sulfone-CALB biocatalyst, the blocking step was performed on the remaining vs. groups to prevent enzyme-support uncontrolled chemical reactions and to alter the enzyme-support physical interactions [57,59,73,74]. Thus, it was possible to analyze whether the different enzyme-support interactions could somehow alter the enzyme features. Blocking agents with different physical and chemical nature were chosen, such as ethylenediamine (EDA) (a small di-cationic molecule), hexylamine (HA) (a mono cationic molecule with a relatively long hydrophobic moiety) and the amino acids Gly (a small cationic/anionic molecule) and Asp (bearing two anionic and a cationic groups). Table 2 shows the effect of the blocking step on the activity of the biocatalysts versus different substrates when using different agents. The activity of the unblocked preparation was considered as reference (100%). The biocatalyst blocked with EDA slightly increased the immobilized enzyme activity, and the other blocking reagents produced a decrease in enzyme activity. The biocatalyst blocked with Asp showed the lowest activity, followed by the one blocked with HA and Gly. Similar incubations of octyl-CALB did not produce significant alterations of the enzyme features. Then, the blocking agent seems to alter the enzyme-support interactions affecting the final enzyme features [57,59,73,74]. The SDS-PAGE analysis of the blocked biocatalysts revealed that most enzyme molecules were covalently immobilized (Figure S1). As this is performed on the immobilized biocatalyst, we can expect that this strategy gives reliable percentage of enzyme molecules non-covalently attached to the support, even if they are very strongly adsorbed [45]. The analysis of the eluents used during the washing steps may also point towards enzyme release, but the detection limit is lower [75].

### 2.3. Activity of CALB Biocatalysts versus the Different Substrates

The activities of the different OC-VS-CALB preparations compared to the octyl biocatalyst versus *p*NPB, triacetin, and (*S*)-methyl mandelate may be found in Table 3. The highest activity was obtained with *p*NPB as substrate for all the biocatalysts, followed by triacetin and (*S*)-methyl mandelate. Using *p*NPB, the activity using the ethylenediamine (EDA) blocked biocatalyst was slightly higher than that of the octyl biocatalyst, while the blocking using Asp and hexylamine decreased the activity in a similar way (by around 20%) and the Gly blocking produced a very slight activity decrease. The activity versus triacetin was the highest for octyl-CALB, while the least active preparations were those blocked with Gly and EDA, with similar activities (decreasing the activity by 30%) with the Asp-blocked biocatalyst being the most active among the vs. biocatalysts. Using the (*S*)-isomer of methyl mandelate, the vs. biocatalysts were quite less active than the octyl biocatalyst (by around 50%) and not very different among them (from 11.1 U/g using the EDA-blocked biocatalyst to 8.7 U/g using the Asp-blocked biocatalyst). These differences among the enzyme specificities of the differently blocked biocatalysts were lower than those previously found using TLL [61].

### 2.4. Thermal Inactivation of CALB Biocatalysts at Different pH Values

**Figure 2:**
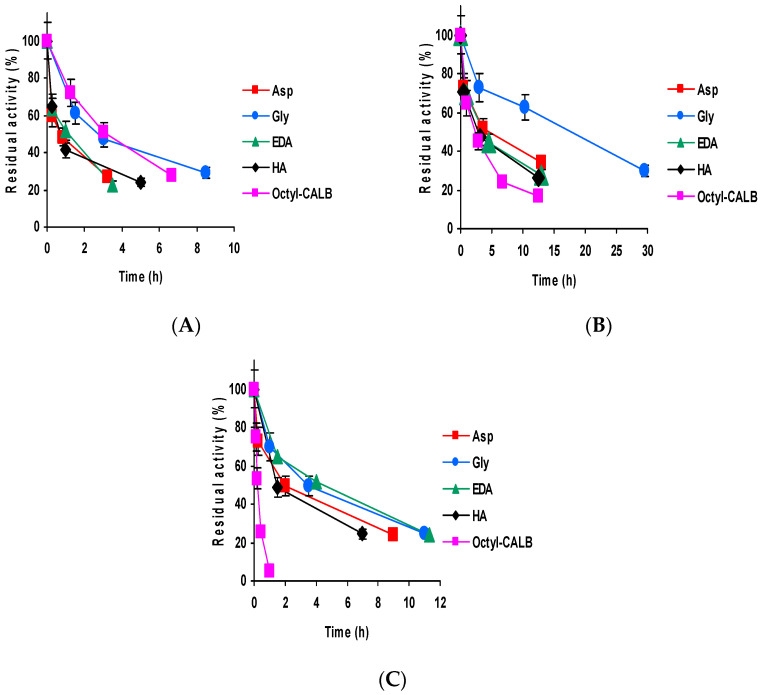
Inactivation courses of CALB biocatalysts. Inactivation conditions: (**A**) 50 mM of sodium acetate at pH 5.0 and 80 °C; (**B**) 50 mM of Tris HCL 50 mM at pH 7.0 and 75 °C; (**C**) 50 mM of sodium carbonate at pH 9.0 and 60 °C. Experiments were carried out as described in Methods.

Figure 2 shows the inactivation courses of CALB biocatalysts at different pH values. At pH 5 (Figure 2A), OC-VS-CALB-Gly and octyl-CALB were the most stable preparations, the other 3 preparations being less stable. At pH 7, the situation was fully different; now all octyl-VS biocatalysts were more stable than the octyl-CALB preparation, with OC-VS-CALB-Gly clearly the most stable one and the other three octyl-VS biocatalysts presenting similar inactivation courses. At pH 9, all VS-octyl biocatalysts were much more stable than the octyl biocatalyst, with very similar stabilities, with the least stable being that modified with HA. The differences in stability caused by the blocking agent using CALB were lower than those found using TLL [61]. These results were similar to those reported using octyl-glyoxyl-CALB [46].

### 2.5. Enzyme Reactivation during the 24 h Incubation at 25 °C Using pNPB as Substrate

To analyze if the CALB biocatalysts could recover part of the lost activity during the thermal inactivation when incubated under mild conditions, as has been previously reported [48,61,76], the enzymatic activities of the partially inactivated biocatalysts were measured after the stress inactivation (leaving the biocatalyst activity decrease to around 75, 50 and 25%) followed by 24 h of incubation at pH 7 and 25 °C (Table 4).

Octyl-VS-CALB-Asp inactivated at pH 5 recovered significant activity even when the inactivation was of only 60% (from 60% to 74%), with the highest recovery of activity being when the biocatalyst was inactivated down to 50% (recovering activity to 70%). When inactivated at pH 7, only a significant activity recovery was observed at the lowest inactivation level (activity went from 73% to more than 90%). After inactivation at pH 9, activity only significantly increased on the highest level of inactivation and not in a very relevant way (from 24% to 30%).

Analyzing the results using octyl-VS-CALB-Gly, some recovery of activity could only be detected when analyzing the most inactivated preparation inactivated at pH 9, and in other cases activity even decreased. Octyl-VS-CALB blocked with ethylenediamine (EDA) increased its activity very significantly after 24 h incubation under mild conditions when inactivated at pH 5 (going from 23 to 63% for the most inactivated biocatalyst), and after inactivation at pH 7 the reaction was not significant using the most inactivated biocatalyst but was very relevant for the other two inactivation levels. After inactivation at pH 9, only the most inactivated biocatalysts recovered a significant percentage of activity (from 24 to 35%). Finally, focusing on octyl-VS-CALB blocked with hexylamine (HA), a significant reactivation was observed when the enzyme was inactivated at pH 5 (e.g., the activity increase from 24 to 45%), remained unaltered when inactivated at pH 7 and recovered some activity when inactivated at pH 9 (e.g., from 25 to 32%).

This dispersion on the effects of the incubation under mild conditions can be explained by the different enzyme-support interactions and the different structures formed by the inactivation under different conditions, as previously reported [61,77].

### 2.6. Alterations of CALB Preparations Specificity during Inactivation under Different Conditions

Different studies have reported that small changes in the lipase immobilization protocol may alter its structure and strongly modify the enzyme specificity [60,61,78,79,80,81]. Then, the activities of different CALB biocatalysts versus three structurally different substrates were analyzed: triacetin, the most similar to the natural substrates of the enzyme (triglycerides); the isomer (*S*)-methyl mandelate; and the synthetic monofunctional p-nitrophenyl butyrate (*p*NPB).

**Figure 3:**
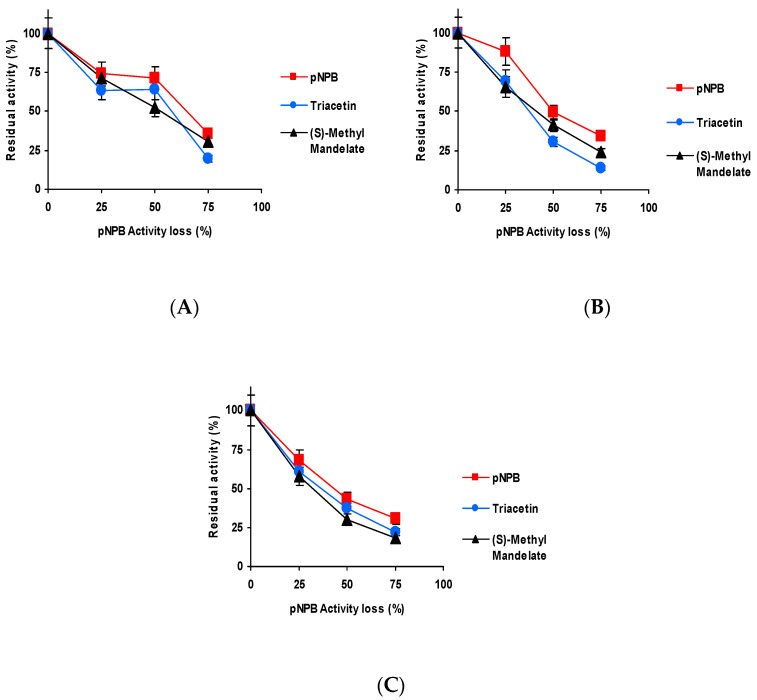
Effect of the inactivation pH on the residual activity versus different substrates of CALB biocatalysts activity blocked with aspartic acid. Inactivation was performed at (**A**) pH 5.0 and 80 °C; (**B**) pH 7.0 and 75 °C; and (**C**) pH 9.0 and 60 °C. The substrates used were: *p*NPB; triacetin; and (*S*)-methyl mandelate. Other specifications may be found in Methods section.

Figure 3 shows the effect of the inactivation pH on CALB biocatalysts blocked with Asp activity versus the three substrates. Differences were shorter than those detected using TLL [61] but were still relevant. When inactivated at pH 5 and 7, the activity was better maintained using *p*NPB as substrate, and the fastest inactivation was observed using triacetin as substrate. At pH 9 (Figure 3C), the situation was slightly different, *p*NPB remained as the substrate that enabled to retain the highest residual activity, but (*S*)-methyl mandelate and triacetin produced similar inactivation courses.

**Figure 4:**
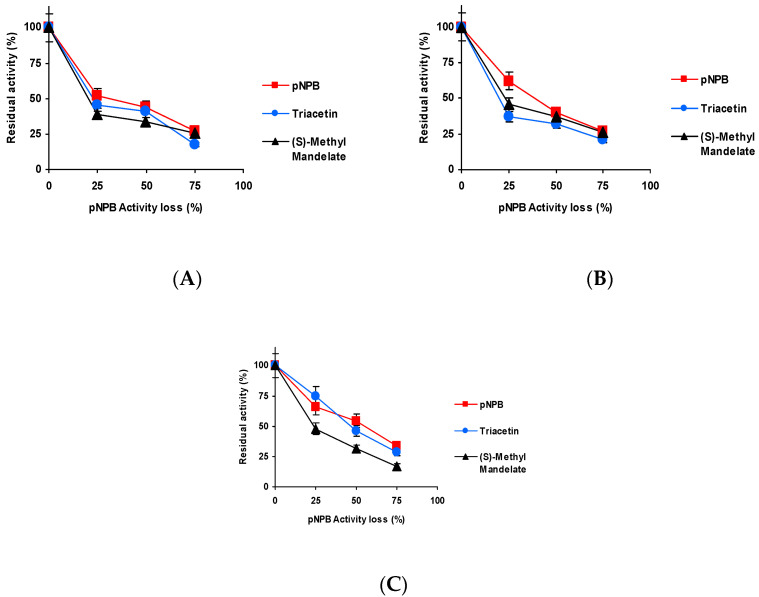
Effect of the inactivation pH on the residual activity versus different substrates of CALB biocatalysts activity blocked with glycine. Inactivation was performed at (**A**) pH 5.0 and 80 °C, (**B**) pH 7.0 and 75 °C and (**C**) pH 9.0 and 60 °C. The substrates used were: *p*NPB, triacetin, and (*S*)-methyl mandelate. Other specifications may be found in Methods section.

The results obtained using the biocatalyst blocked with Gly may be found in Figure 4. Although again slightly better residual activity was observed using *p*NPB, only at pH 9 is the inactivation clearly faster using (*S*)-methyl mandelate as substrate.

**Figure 5:**
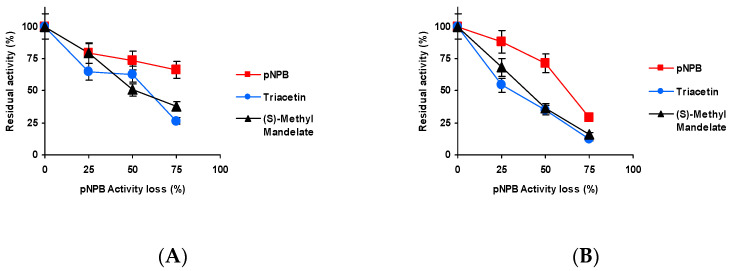

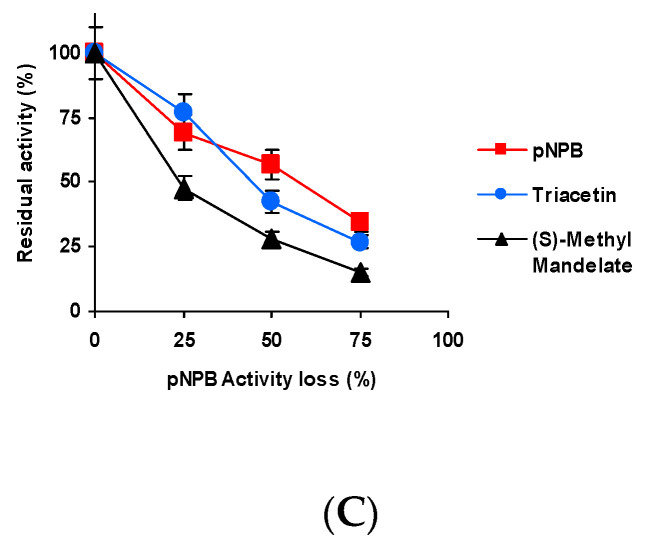
Effect of the inactivation pH on the residual activity versus different substrates of CALB biocatalysts activity blocked with EDA. Inactivation was performed at (**A**) pH 5.0 and 80 °C, (**B**) pH 7.0 and 75 °C and (**C**) pH 9.0 and 60 °C. The substrates used were: *p*NPB; triacetin; and (*S*)-methyl mandelate. Other specifications may be found in Methods section.

Figure 5 shows the results using the biocatalysts blocked with ethylenediamine (EDA). The differences with the different substrates are more significant for this biocatalyst. Again, the slowest inactivation is observed using *p*NPB, only at pH 9 is the inactivation clearly more rapid using (*S*)-methyl mandelate than using triacetin.

**Figure 6:**
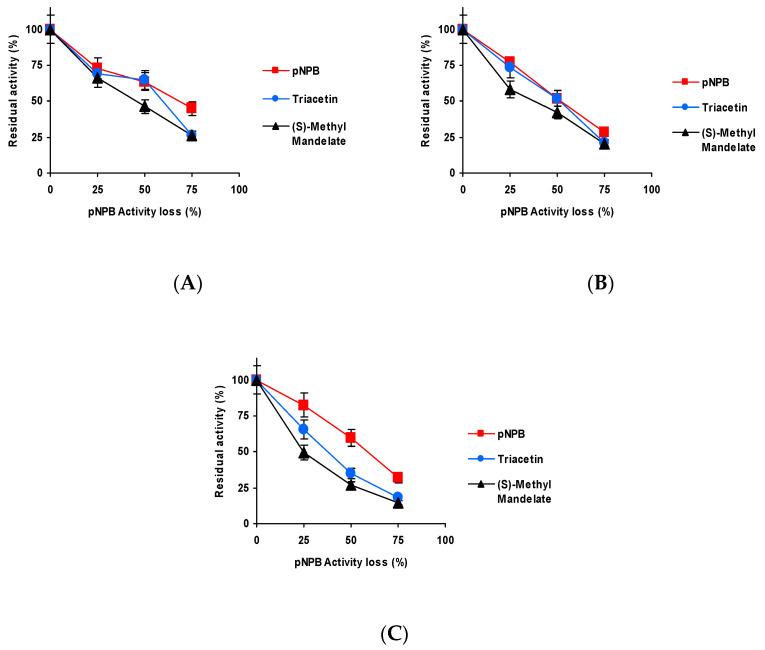
Effect of the inactivation pH on the residual activity versus different substrates of CALB biocatalysts activity blocked with hexylamine. Inactivation was performed at (**A**) pH 5.0 and 80 °C, (**B**) pH 7.0 and 75 °C and (**C**) pH 9.0 and 60 °C. The substrates used were: *p*NPB; triacetin; and (*S*)-methyl mandelate. Other specifications may be found in Methods section.

Figure 6 shows the results utilizing the biocatalysts blocked with hexylamine. Differences in the inactivation courses are not very large, but again inactivation is slower using *p*NPB (except at pH 7, where it is almost identical to that using triacetin), and the fastest is always that observed using (*S*)-methyl, with the clearest differences being when the inactivation was performed at pH 9.

Thus, even though differences are not so large as in the case of TLL [61], still there are some differences in the inactivation courses depending on the substrate used to determine the enzyme activity and blocking agent.

### 2.7. Fluorescence Studies of the Different CALB Biocatalysts

We tried to couple the changes in the functional properties to structural changes in the enzyme. The fluorescence spectroscopy of tryptophan residues might provide useful information of the biomolecule structure [82]. This analysis is mainly related to the solvent exposure of Trp residues and is highly susceptible to the polarity of the microenvironment [83]. The amino acid sequence of CALB shows 5 tryptophan residues in its structure (W52, W65, W104, W113 and W155) [65].

**Figure 7:**
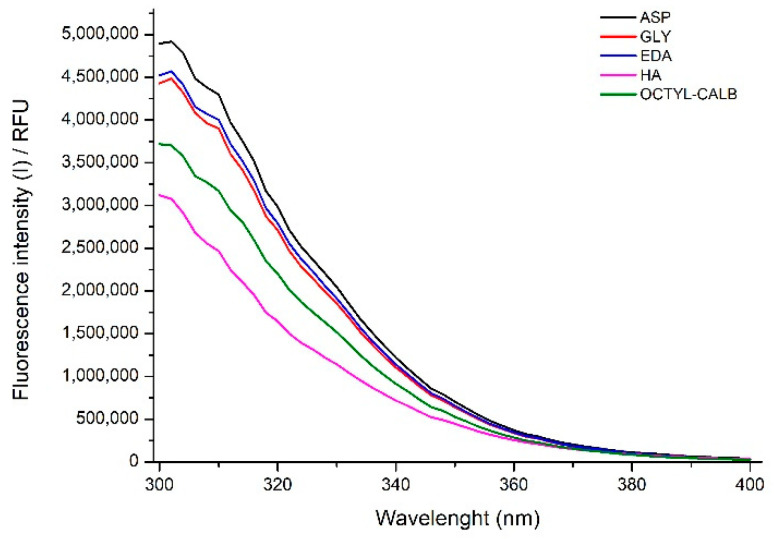
Intrinsic protein spectra of CALB biocatalysts blocked with different blocking reagents. Other specifications may be found in Methods section.

**Figure 8:**
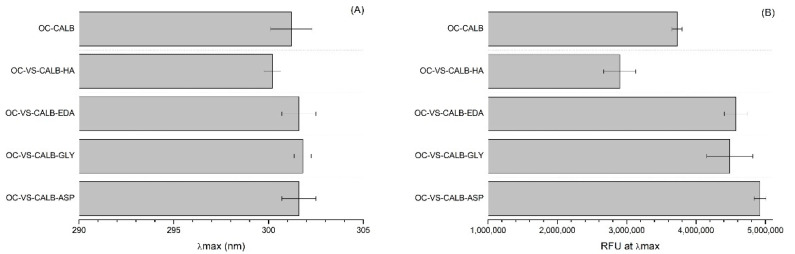
Intrinsic protein fluorescent of OC-VS-CALB biocatalysts blocked with different blocking reagents and octyl-CALB as reference. (**A**) Wavelength at the maximum fluorescence intensity (λmax). (**B**) Fluorescence intensity at λmax when samples were excited at 280 nm. RFU = relative fluorescence units.

Prior to fluorescence analysis, all biocatalyst samples (non-inactivated and partially inactivated ones) were kept in 50 mM Tris-HCl buffer pH 7 for at least one week to ensure that the differences were produced by the previous incubations [61]. This technique permits us to determine the exposition of the Trp to the water, and this exposition is expected to produce a decrease in the fluorescence emission and a red shift of the maximum emission wavelength (towards higher values). Using enzymes immobilized on octyl supports, this may be not so obvious as Trp interacting with the octyl groups could remain with similar intensities to being inside the core of the protein or even increase fluorescence and decrease emission wavelength. The situation can be also altered in our current samples as the different blocking agents have different polarity. The intrinsic protein fluorescence spectra are shown in Figure 7, while Figure 8 shows wavelength where the maximum emission intensity is observed and this maximum. It is obvious that the spectra profiles are different for the different immobilized enzymes. Hexylamine (HA), a hydrophobic blocking agent, produces a decrease in the emission wavelength but a high decrease in the intensity, with this contradictory trend related to the environment of the Trp groups compared to that in the octyl support. The other 3 blocking agents have the opposite effect, increasing the wavelength and the emission intensity.

Again, this is contradictory. However, these results are the balance between the conformational changes that the enzyme suffers due to enzyme-support interactions and the alterations of the Trp environments caused by the immobilization/blocking, making hard to properly understand the meaning. The changes induced by the inactivation in these fluorescence features during enzyme inactivation must be related to the way the Trp groups alter its position, as in this case, where the support remains unchanged.

**Figure 9:**
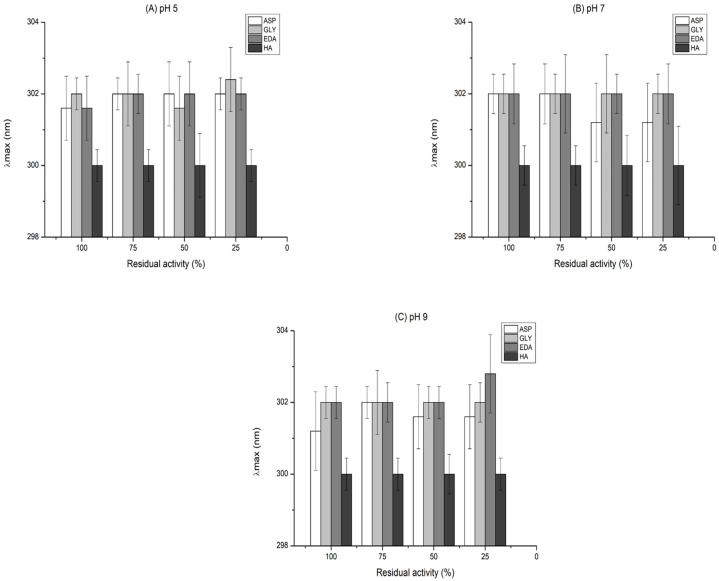
Intrinsic fluorescence (λmax) of immobilized CALB biocatalyst blocked with different reagents and inactivated at different pH values pH 5 (**A**), pH 7 (**B**), and pH 9 (**C**). From the intrinsic fluorescence spectra, (λmax) were plotted for the different CALB at different inactivation degrees. The residual activity means the activity exhibited by the CALB after being incubated at a certain time at different pH. The residual activity at 100% indicates that they are fully active biocatalysts.

**Figure 10:**
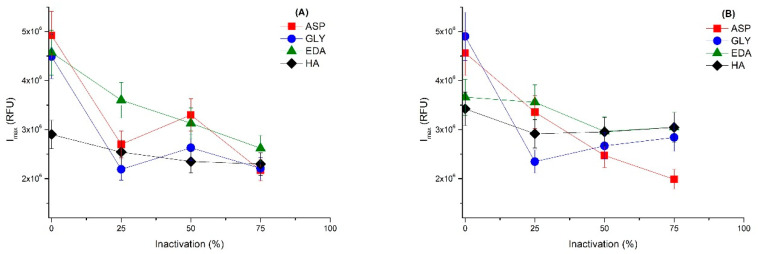

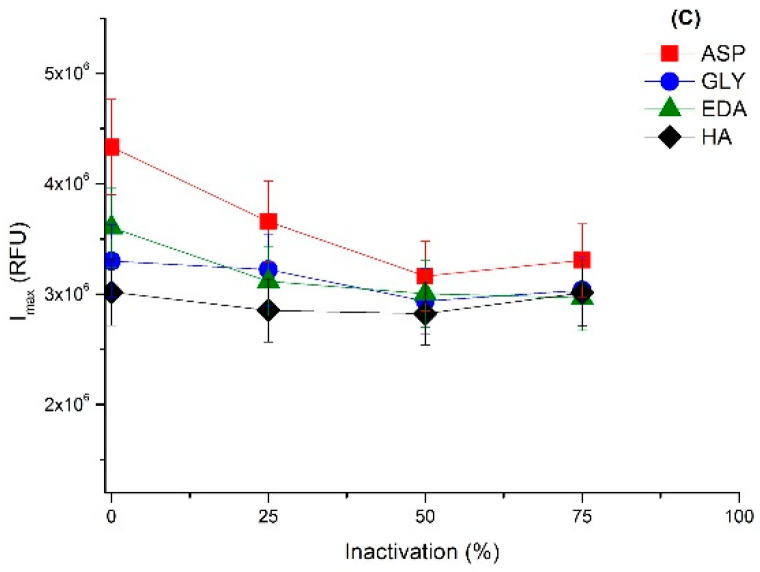
Changes of the intrinsic fluorescence (Imax at λmax) during the different biocatalysts inactivation. Imax at λmax was plotted for the different CALB at different inactivation degrees. The inactivation percentage is the activity lost by the different CALB biocatalysts after being incubated at a certain time at different pH under inactivating temperatures. Conditions: (**A**) pH 5; (**B**) pH 7; and (**C**) pH 9. RFU = relative fluorescence units.

Analyzing fluorescence spectra when the enzyme was inactivated under each pH condition, Figure 9 shows the wavelength at the maximum fluorescence intensity (λmax), while Figure 10 shows the changes in the intensity of this maximum. HA had no maximum wavelength variation at any inactivation percentage for all pHs. At pH 7, only in the biocatalyst blocked with Asp was there a variation of maximum emission wavelength. The other biocatalysts inactivated at different pH maintained this parameter with slight variations for all inactivation percentages.

Figure 10 shows that the inactivation of the different biocatalysts initially produces the expected decrease in the emission intensity when the enzyme structure is less compact. However, in some instances, after the initial decrease it is possible to observe an increase, which could be consequence of the interactions of the Trp with the octyl groups after enzyme conformational changes. The intensity and direction of these emission intensity changes depend on the pH and the biocatalysts, confirming that the conformational changes induced by the inactivation conditions depend on the inactivating pH value and support blocking.

## 3. Materials and Methods

### 3.1. Materials

Liquid CALB (the isoform B from *Candida antarctica*) (5.07 mg of protein/mL) was kindly donated by Novozymes (Copenhagen, Denmark). Octyl-Sepharose^®^ CL-4B beads were purchased from GE Healthcare (Contagem, Brasi). Triacetin, p-nitrophenyl butyrate (*p*-NPB), L-aspartic acid, hexyl amine (HA), ethylenediamine (EDA), divinyl sulfone (DVS), glycine and (*S*)-methyl mandelate were from Sigma Aldrich (São Paulo, Brazil). All other reagents and solvents were of analytical grade. Bradford’s method [84] was used to quantify the protein concentration with bovine serum albumin as reference.

### 3.2. Methods

All experiments were performed in triplicate; the results are reported as their mean values and the standard deviation.

### 3.3. Preparation of Octyl-Vinyl Sulfone Support (Octyl-VS)

The support activation was performed according to the procedure reported by Albuquerque et al. [56], with some modifications. A solution was prepared with 2.5 mL of DVS and 66.6 mL of 333 mM sodium carbonate at pH 11.5 (to a final concentration of 0.35 M) and stirred with a magnetic stirring bar until the medium turned homogeneous. Then, 10 g of octyl-agarose beads were added and left under gentle stirring for 2 h. After that, the support was filtered by vacuum, washed 5 times with 10 volumes of distilled water and stored at 4 °C. After the third washing activity was not detected on the washing waters, we preferred to add two additional washings to ensure the full elimination of all unbound components of the crude.

### 3.4. Immobilization of CALB on Octyl-VS Support

CALB was immobilized by interfacial activation using a protein loading of 1 mg/mL [42]; 1 gram of support was added per 10 mL of enzyme solution (0.1 mg/mL enzyme in 5 mM sodium acetate at pH 5.0) at 25 °C. The immobilization was performed under gentle mechanical stirring, and the activities of supernatant and suspension were measured using p-NPB as substrate (the enzyme was fully stable under these conditions). After immobilization, the biocatalyst was vacuum filtered, washed with distilled water and resuspended in 50 mM sodium bicarbonate at pH 8.0 and 25 °C for 4 h to favor the enzyme-support covalent reaction [56]. After the third washing activity was not detected on the washing waters, we preferred to add two additional washings to ensure the full elimination of all unbound components of the crude. Then, the octyl-VS biocatalyst was recovered by vacuum filtration and blocked with different blocking agents [56,60], as described below.

### 3.5. Blocking of the Octyl-VS-CALB Biocatalysts

As a reaction endpoint, the enzyme immobilized in octyl-VS was incubated in 2 M of different nucleophiles (EDA, HA, Gly or Asp) at pH 8.0 and 25 °C for 24 h (10 mL of blocking solution per 1 g of biocatalysts) to block the remaining vinyl sulfone groups in the support [56]. Finally, the covalently immobilized and blocked biocatalysts were vacuum filtered and washed 5 times with 10 volumes of distilled water and stored at 4 °C.

### 3.6. Determination of Enzyme Activity versus Different Substrates

One enzyme activity unit (U) was defined as one µmol of substrate hydrolyzed per minute under the described conditions.

#### 3.6.1. Hydrolysis of p-NPB

The enzymatic activity was quantified by determining the increase in absorbance at 348 nm (isosbestic point, ε under these conditions is 5150 M^−1^ cm^−1^) during 90 s produced by the p-nitrophenol released in the hydrolysis of p-NPB [85] using an Evolution 220 UV-Visible Spectrophotometer (Thermo Fisher Scientific, Miami, FL, USA). The reaction was started by adding 50 μL of the sample (free enzyme solution or, immobilized enzyme suspension) in 2.5 mL of 25 mM sodium phosphate at pH 7.0 and 25 °C containing 50 μL of p-NPB solution (at a concentration of 50 mM, dissolved in acetonitrile) under magnetic stirring and temperature control.

#### 3.6.2. Hydrolysis of Triacetin

Samples of 0.05 g of wet biocatalysts (submitted to thermal inactivation or not) were added to 3–4 mL of 50 mM triacetin in 50 mM sodium phosphate at 25 °C and pH 7.0. The mixture was continuously stirred using a rotating mixer (Basic Rotor K45-3220, Kasvi, Brazil). The reaction product was measured and quantified by HPLC (Agilent Technologies 1260 Infinity (Santa Clara, CA, USA) using a Zorbax SB-C18 column (15 cm × 0.46 cm) (Agilent Technologies, Santa Clara, CA, USA) with an IR detector injecting samples of 20 μL. A solution of 55% acetonitrile/45% Milli-Q was used as a mobile phase with a flow of 0.6 mL/min. The column was thermostated at 25 °C, and the IR detector temperature was set at 35 °C. Under these reaction conditions, the enzyme product, 1,2 diacetin, suffers acyl migration, and a mixture with 1,3 diacetin is obtained, but both products co-eluted under these conditions [80]. The retention times of the compounds were 2.8 min for diacetins and 4 min for triacetin. Conversions between 15 and 20% were utilized to estimate the initial reaction rates.

#### 3.6.3. Hydrolysis of (S)-Methyl Mandelate

Samples of 0.05 g of wet biocatalysts (submitted to thermal inactivation or not) were added to 3–4 mL of 50 mM triacetin in 50 mM sodium phosphate buffer at 25 °C and pH 7.0. The mixture was continuously stirred using a rotating mixer (Basic Rotor K45-3220, Kasvi, Brazil). The product was quantified in an HPLC, Agilent Technologies 1260 Infinity (Santa Clara, CA, USA). The column was a Zorbax SB-C18 column (15 cm × 0.46 cm) (Agilent Technologies, Santa Clara, CA, USA) at 25 °C, and a solution of 40% acetonitrile/60% Milli-Q was used as mobile phase with a flow of 1 mL/min. The compounds were determined with UV/VIS detector at 230 nm, by injecting reaction samples of 20 μL and the retention times were about 1.3 min for mandelic acid and 2.8 min for methyl mandelate. Conversions between 15 and 20% were used to calculate the initial reaction rates.

### 3.7. Thermal Inactivation of the Different CALB Biocatalysts at Different pH Values

The inactivation of the biocatalysts was measured at the indicated pH values at different temperatures to select the inactivation conditions. Due to different stabilities, selecting different temperatures at each pH value was required to have reliable inactivation courses. Thus, the biocatalysts were incubated in 50 mM sodium acetate at pH 5.0 and 80 °C; 50 mM Tris HCl at pH 7.0 and 75 °C (phosphate was avoided due its adverse effects on immobilized lipases stabilities) [86]; or 50 mM sodium carbonate at pH 9.0 and 60 °C. Periodically, samples were withdrawn, and their residual activities were measured using the p-NPB assay described previously. Residual activities were calculated as the percentage of the initial activities in determined moment. The inactivation courses were stopped removing the biocatalyst from the reaction media (time was different for each biocatalyst and inactivation condition). The biocatalysts were vacuum filtered when their residual activity was 75%, 50% and 25%. All these biocatalysts were washed with 50 mM Tris buffer at pH 7, stored at 25 °C for 24 h to enable any reactivation [61,78,87,88,89] and then stored at 4 °C to analyze their activities versus different substrates and fluorescence spectra.

### 3.8. Fluorescence Studies of the Different Immobilized CALB Preparations

The intrinsic fluorescence of immobilized CALB was measured in 96-well solid black polystyrene microplates; 100 μL of 1:10 (*w*:*v*) suspension of immobilized CALB containing 10 μg of protein in 100 mM Tris-HCl buffer at pH 7 were added to each well. The study was performed by exciting each well at 280 nm with a 2 nm slit and recording the emission spectrum at 300–400 nm using a microplate reader Synergy Mx, BioTek^®^ with the software Gen5. The maximum fluorescence intensity (Imax) and the wavelength at maximum intensity (λmax) were calculated by subtracting the spectra of the empty carriers from the spectra of the samples with the immobilized CALB. The raw fluorescence data obtained from the fluorometer were analyzed using Origin 8 software.

### 3.9. SDS-PAGE Analysis

SDS-polyacrylamide gel electrophoresis (SDS-PAGE) was carried out according to Laemmli [90], using 5% polyacrylamide stacking gel and 12.5% polyacrylamide resolving gel. In the case of free enzyme, the solutions were diluted in rupture buffer (4% SDS and 0.2 M dithiothreitol—DTT) to the final concentration of 1 mg of protein/mL. For immobilized CALB, 100 mg were resuspended in 200 μL of rupture buffer. Then, this suspension was boiled for 10 min, enabling the release of all enzyme molecules not covalently attached to the support [45].

## 4. Conclusions

The use of octyl-VS to immobilize CALB permits us to avoid enzyme release that can occur in the octyl biocatalyst. The effects on enzyme features depend on the blocking agent. At pH 7 and mainly at pH 9, all biocatalysts become more stable than the octyl biocatalysts, although those blocked using Gly usually was the most stable. At pH 5, only the blocking with Gly produced biocatalyst with a stability similar to that of the octyl biocatalysts, decreasing the stability for the other biocatalysts. The activity of the vs. usually decreased compared to that of the octyl biocatalysts, although the activity versus *p*NPB of the biocatalyst blocked with EDA was slightly higher. This was correlated to different fluorescence spectra, suggesting different conformations of the enzyme depending on the blocking agent.

The inactivation courses followed by measuring the residual activities using three different substrates show certain differences, but in any case they are not as significant as those found studying TLL; the activity decrease is similar for all the substrates, although they depend on the blocking agent. The changes in fluorescence parameters during inactivation of the different biocatalysts were not very dissimilar among the biocatalysts, with exception of the biocatalyst blocked using Asp.

Thus, the immobilization of CALB on acyl-VS may become an adequate strategy to immobilize the enzyme, but the blocking agent must be selected in each specific case to maximize the benefits.

## Figures and Tables

**Table 1:** Effect of incubation time (0–4 h) at pH 8 and 25 °C on the activity of OC-VS-CALB biocatalyst.

Biocatalyst	Relative Activity (%)
OC-VS-CALB (after immobilization)	100 ± 4.2
OC-VS-CALB (0.1 h of incubation)	88.7 ± 3.3
OC-VS-CALB (after 4 h of incubation)	95.1 ± 3.1

**Table 2:** Effect of the different blocking agents on OC-VS-CALB biocatalyst. The activity was measuring versus pNPB. The biocatalysts have been incubated for 24 h at pH 8 after incubation step.

Biocatalyst	Relative Activity (%)
OC-VS-CALB (unblocked)	100 ± 3.7
OC-VS-CALB Blocked with ASP	75.9 ± 1
OC-VS-CALB Blocked with GLY	88.5 ± 4.7
OC-VS-CALB Blocked with EDA	104.6 ± 2
OC-VS-CALB Blocked with HA	77.6 ± 4.7

**Table 3:** Mass activity (U/g) of different immobilized CALB biocatalysts with 50 mM (*S*)-methyl mandelate (pH 7.0, 25 °C), 50 mM of triacetin (pH 5.0, 25 °C) or 1 mM of *p*NPB (pH 7.0, 25 °C). Experiments were performed as described in Methods.

Activity (U/g of Biocatalyst)				
CALB Preparations	Blocking Agent	*p*NPB	Triacetin	(*S*)-Methyl Mandelate
OC-CALB	-	340.6 ± 22.8	22.2 ± 0.4	18.8 ± 0.4
OC-VS-CALB	ASP	278.7 ± 22.7	18.6 ± 0	8.7 ± 0.3
	GLY	323.2 ± 7.1	15.4 ± 0.5	9.4 ± 0.2
	EDA	367.9 ± 5.6	15.6 ± 0.5	11.1 ±0.2
	HA	269.2 ± 9.3	17.7 ± 0.2	8.9 ± 0.3

**Table 4:** Residual activity of different immobilized CALB biocatalysts in the inactivation courses at pH 5.0 and 80 °C, pH 7.0 and 75 °C and pH 9.0 and 60 °C. The activities were taken during the inactivation and after 24 h of incubation at pH 7.0 and 25 °C using pNPB as substrate. Means in the same line and blocking agent sharing the same letter (a or b) are not statistically different according to the Tukey test (*p* > 0.05).

RESIDUAL ACTIVITY (%)									
NAME OF THE BIOCATALYST		ASP		GLY		EDA		HA	
		INACTIVATION	AFTER 24 h	INACTIVATION	AFTER 24 h	INACTIVATION	AFTER 24 h	INACTIVATION	AFTER 24 h
pH 5	75%	60 ± 1.7 ^a^	74 ± 0.2 ^b^	61 ± 1.7 ^a^	52 ± 0.5 ^b^	63 ± 3.4 ^a^	79 ± 0 ^b^	65 ± 5.2 ^a^	73 ± 1.7 ^a^
	50%	48 ± 3.1 ^a^	70 ± 3.2 ^b^	47 ± 2.1 ^a^	45 ± 2.1 ^a^	52 ± 5.7 ^a^	74 ± 0.2 ^b^	41 ± 2.8 ^a^	63 ± 0.2 ^b^
	25%	27 ± 3 ^a^	35 ± 0.2 ^a^	29 ± 0.8 ^a^	28 ± 0.4 ^a^	23 ± 3.6 ^a^	64 ± 0.2 ^b^	24 ± 0.7 ^a^	45 ± 0.9 ^b^
pH 7	75%	73 ± 1.1 ^a^	91 ± 1.2 ^b^	73 ± 2.2 ^a^	62 ± 1.1 ^b^	69 ± 3.8 ^a^	88 ± 2.3 ^b^	71 ± 3 ^a^	75 ± 2.8 ^a^
	50%	52 ± 0.8 ^a^	51 ± 3.1 ^a^	63 ± 3.2 ^a^	39 ± 0.7 ^b^	44 ± 2.4 ^a^	69 ± 0.2 ^b^	47 ± 2.3 ^a^	48 ± 1.8 ^a^
	25%	34 ± 2.5 ^a^	35 ± 1.4 ^a^	29 ± 0.6 ^a^	26 ± 0 ^a^	28 ± 2.4 ^a^	29 ± 0.8 ^a^	26 ± 2.3 ^a^	227 ± 1.2 ^a^
pH 9	75%	73 ± 1 ^a^	69 ± 2.3 ^a^	70 ± 1.9 ^a^	67 ± 1.2 ^a^	65 ± 4.4 ^a^	67 ± 1 ^a^	65 ± 0 ^a^	83 ± 2.1 ^b^
	50%	49 ± 0 ^a^	43 ± 2 ^b^	49 ± 2.6 ^a^	52 ± 0.9 ^a^	52 ± 2.3 ^a^	47 ± 1.6 ^a^	49 ± 0 ^a^	60 ± 1.4 ^b^
	25%	24 ± 1 ^a^	30 ± 0.8 ^b^	25 ± 1.9 ^a^	34 ± 0.6 ^b^	24 ± 1.5 ^a^	35 ± 0.3 ^b^	25 ± 0.8 ^a^	32 ± 1.8 ^b^

## Data Availability

Data can be obtained from the authors upon request.

## Supplementary Materials

The following are available online at https://www.mdpi.com/article/10.3390/ijms232214268/s1.
