# Supplementary material for: Immobilization of Lipase B from Candida antarctica in Octyl-Vinyl Sulfone Agarose: Effect of the Enzyme-Support Interactions on Enzyme Activity, Specificity, Structure and Inactivation Pathway
Source: Int J Mol Sci. 2022 Nov 17;23(22):14268. doi: 10.3390/ijms232214268 (PMC9697615; doi:10.3390/ijms232214268)
Supplement: Supplementary file 1 [file ijms-23-14268-s001.zip › ijms-1983858-supplementary.pdf]

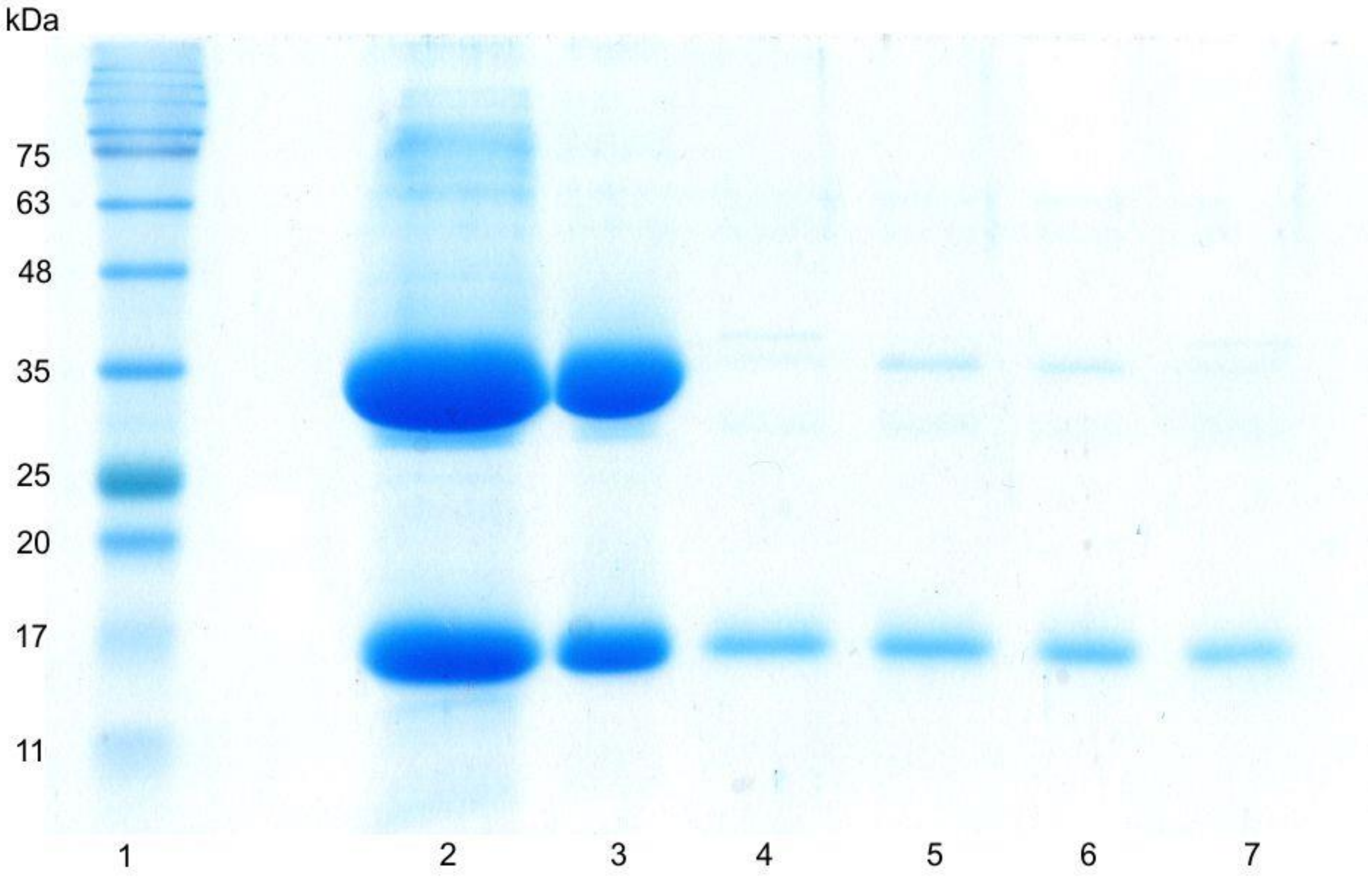

Figure S1 SDS-PAGE analysis of different preparations of CALB. Experiments were performed as described in Methods. Lane1: weight molecular marker. Lane 2: free CALB (1 mg/ml). Lane 3: OC-CALB. Lane 4: OC-VS-CALB-Asp. Lane 5: OC-VS-CALB-Gly. Lane 6: OC-VS-CALB-EDA. Lane 7: OC-VS-CALB-HA.
